# Prevalence and Associated Risk Factors of Bronchial Asthma in Children in Santo Domingo, Dominican Republic

**DOI:** 10.7759/cureus.2211

**Published:** 2018-02-20

**Authors:** Stephanie G Mejias, Kamleshun Ramphul

**Affiliations:** 1 Department of Pediatrics, Robert Reid Cabral Children's Hospital Affiliated to the University Iberoamericana Unibe School of Medicine; 2 Department of Pediatrics, Shanghai Xin Hua Hospital Affiliated to Shanghai Jiao Tong University School of Medicine, Shanghai, People's Republic of China

**Keywords:** asthma, pediatrics, santo domingo

## Abstract

Background

Bronchial asthma is an important health problem worldwide. There is insufficient data on the prevalence of bronchial asthma among school children in Santo Domingo, Dominican Republic.

Objective

The objective of this study is to assess the prevalence of asthma and its related risk factors among school children in Santo Domingo, Dominican Republic.

Materials and methods

A cross-sectional study using a modified questionnaire was conducted in Santo Domingo among 600 children aged three to 11 eleven years. The prevalence of asthma and its associated risk factors such as birth order, family history of asthma, family history of allergy, exposure to pets at home, exposure to tobacco smoke, and source of fuel used at home were collected. The relevant data collected was analyzed using the Statistical Package for the Social Sciences (SPSS) 24.0. (IBM Corp., Armonk, NY) software.

Results

The prevalence of asthma was found to be 22.0%. Age, family history of asthma, family history of allergy, exposure to tobacco smoke, and birth order showed statistical significance. The source of fuel used at home, gender, and exposure to pets were not statistically significant to be considered as risk factors associated with asthma in the population studied.

Conclusion

With an asthma prevalence of 22.0% in the pediatric population, the Dominican Republic has one of the highest national rates of asthma in the pediatric population in Latin America. Proper education, screening, and prevention can help lower the burden of this disease economically and socially.

## Introduction

Asthma is a chronic respiratory disorder involving the airways and is characterized by variable and recurring symptoms. It is usually associated with airflow obstruction, inflammation, and bronchial hyper-responsiveness [1]. Asthma is one of the most important chronic diseases worldwide and it affects individuals of all ages [2]. Patients usually present with occasional episodes of asthma attacks, breathlessness, wheezing, and chest tightness [3]. The allergen or precipitating factor causes airway hyper-responsiveness that leads to narrowing of the passages, causing obstruction of airflow [4].

There is a huge variation in the incidence rate of asthma geographically. In African countries the incidence ranges from 18% in Kenya to 20% in South Africa [5], while in the Asian continent, Pakistan [6] has an incidence rate of 15.8% and India, 5.3%. Recent studies have shown that in most of the Latin America countries, the prevalence of asthma varied from 2.6% in Guatemala [7] to 33.1% in Peru [8]. In 2012 Caban-Martinez et al. found that the overall prevalence of asthma in the general population of the Dominican Republic was 14.2% [9], and in 2017 Sun et al. noted a prevalence rate of 25.7% [10] in the pediatric population, though the sample size in the latter research was only 74 participants recruited at a medical outreach clinic in Santo Domingo, Dominican Republic. 

Asthma is one of the most prevalent chronic conditions in the Dominican Republic and its impact and burden on society and children’s development continues to rise [11]. The aim of this study is to evaluate the prevalence of childhood asthma and its associated risk factors over a larger sample size in Santo Domingo, Dominican Republic. This will provide a more reliable baseline data on the prevalence of asthma in the pediatric population in the Dominican Republic, which in turn can help improve policies, screening protocols, and guidelines for asthma detection and control.

## Materials and methods

A descriptive cross-sectional study was conducted involving 624 randomly selected children in Santo Domingo, Dominican Republic from October 2017 to December 2017. The sample was evenly divided into males and females and then further divided into four age groups ranging from three to 11 years of age. Six hundred forms that were properly filled and returned were considered for our study, giving a response rate of 96.2%.

Prior permission was obtained from the Dean of the school to allow students to participate. Teachers were given a quick orientation of how to help in the data collection. A questionnaire based on the International Study on Allergy and Asthma [12] was set up and some modifications were made to meet the requirements of the study. The questionnaires were filled in by the parents, and any questions they had were answered by phone and a contact number was listed on each form. The questionnaire was written in both English and Spanish.

Diagnosis of asthma in the children was made according to the guidelines of the American Thoracic Society for the diagnosis of asthma [13]. The first part of the questionnaire focused on the presence and prevalence of wheezing in the children and other atopic conditions such as rhinitis and rashes and their frequency over the last 12 months. The second part consisted of the major risk factors, which included exposure to tobacco smoke at home, presence of any pets, family history of asthma, family history of allergy, type of fuel source used at home, and the birth order.

The collected data was entered into a Microsoft Excel spreadsheet and analyzed using the Statistical Package for the Social Sciences (SPSS) version 24.0. (IBM Corp., Armonk, NY) software package for Windows. Chi square test was used to analyze for associations between categorical variables. Statistical significance was set at p < 0.05.

Ethical clearance was obtained from the school boards, written consent was obtained from the parents of the children, and assent was obtained from the children. Privacy and confidentiality of all participants were maintained.

## Results

Six hundred students were considered for the study, of which 300 were males (50%) and 300 females (50%). In this study, 132 participants were diagnosed with asthma and the prevalence was calculated as 22.0%. Fifty-nine males (19.7%) and 73 females (24.3%) were diagnosed with asthma. No statistical significance was found between gender and the prevalence of asthma in our study. Fourteen children aged between three and five years (9.3%), 33 aged between five and seven years (22.0%), 43 aged between seven and nine years (28.6%), and 42 children between nine and eleven years (28.0%) were diagnosed with asthma in Santo Domingo and this was statistically significant (p<0.05).

Among the 132 children diagnosed with asthma, 30 had a family history of asthma and 43 had a family history of allergy and these two findings were both statistically significant (p<0.05). Sixty children diagnosed with the disease had also been exposed to tobacco smoke while 72 children with asthma were not exposed and this showed statistical significance (p<0.05). Forty-two of the 132 children were first-born children, 68 were second-born, and 22 were the third or higher child in the family. A statistical significance was found (p<0.05) between the birth order and the prevalence of asthma.

In this study we found out that 48 of the asthmatics also had at least one pet at home but no statistical significance was found. Forty-four children with asthma were exposed to gas as a fuel source at home in the kitchen while 88 had an electric source in the kitchen for cooking. No statistical significance was noted in both cases.

The frequencies of the symptoms seen in the asthmatics are shown in Table 1 and the risk factors in Table 2. In this study 98 children (74.2%) had one to three episodes of wheezing over the last 12 months and 34 (25.8%) had more than three. We found that 103 children (78.0%) also suffered from rhinitis and 93 (70.5%) were diagnosed with atopic rashes over the last year.

**Table 1:** Presentation of symptoms in the asthmatics over the last 12 months.

Condition	Prevalence
*Wheezing Episode*	
One to three episodes	98 (74.2%)
Four or more episodes	34 (25.8%)
*Rhinitis*	
Present	103 (78.0%)
Absent	29 (22.0%)
Rashes	
Present	93 (70.5%)
Absent	39 (29.5%)

**Table 2:** Associated factors and the risk of asthma in children from Santo Domingo, Dominican Republic.

Associated factor	Asthma (n=132)	Non-asthma (n=468)	P value
Sex			
Male	59	241	0.168
Female	73	227	
Age			
Three to five	14	136	0.00102
Five to seven	33	117	
Seven to nine	43	107	
Nine to eleven	42	108	
Birth order			
First child	42	95	0.01612
Second child	68	320	
Third child or more	22	53	
Source of fuel used at home​​​​​​​			
Gas	44	200	0.0521
Electric	88	268	
Family history of asthma​​​​​​​			
Present	30	28	<0.01
Absent	102	440	
Family history of allergy​​​​​​​			
Present	43	69	<0.01
Absent	89	400	
Pets at home			
Yes	48	173	0.899
No	84	295	
Exposure to tobacco smoke at home			
Yes	60	133	<0.01​​​​​​​
No	72	335	

## Discussion

The prevalence of asthma in our study for the pediatric population in Santo Domingo, Dominican Republic was 22.0%. It is different from the study conducted by Sun et al. in 2017 for the same population. The sample size, 600 children in our study and 74 children in their study, and the place where the volunteers were recruited, i.e. a school for our study and a clinic for their study, are the possible reasons for the discrepancy noted in the results. The prevalence of 22.0% in the pediatric population is higher than that in the general population (14.2%, Caban-Martinez et al., 2012). However, the finding of 22.0% is very similar to the range in the pediatric population of many Latin American countries such as Honduras (18.3%), Panama (20.5%), Costa Rica (23.2%), and Cuba (30.9%). The environmental exposure and the genetic predisposition in such regions can be some of the major factors for the higher prevalence rate compared to populations of different ethnic backgrounds. In India, for the same ethnic population, the prevalence varied from 7.59% in a less polluted region [14] to 19.34% in a heavy traffic region [15].

Statistical significance was found between birth order and the prevalence of asthma. IL13 gene polymorphism on serum IgE has been associated with birth order [16] and this can be one of the major reasons for the difference observed in our study. Family history of asthma and allergies showed statistical significance in our study. Asthma is a multifactorial condition and recent advances in molecular biology and genetics have strongly suggested that it can be a hereditary disorder based on several factors, including genes [17, 18]. Multiple genetic predispositions have been found in Hispanic populations including the role of PDE4D and PDE4 inhibitors, upon which medications have even been developed for asthma [19].

The presence of at least one smoker in the family showed statistical significance in our study. Passive smoking can be a major risk factor to hyper-responsiveness of airways. Exposure to environmental tobacco smoke (ETS) can lead to poor asthma control in children causing frequent exacerbations and hospitalizations [20-23]. Various studies have shown that exposure of children to tobacco smoke can predispose them to a higher incidence of other pulmonary symptoms (wheezing, asthma, cough, pneumonia, impaired pulmonary function, chronic bronchitis) [24-26] as well as adenoid hypertrophy [27], tonsillitis, and sore throats [28]. Liem et al. showed that parents with a child diagnosed with asthma were unlikely to quit smoking or smoke outside [29]. However, a proper education of the risk factors associated with second hand smoking and asthma and the benefits of protecting their asthmatic children from tobacco exposure should be done in Santo Domingo as the ethnic background of their study and the population in the Dominican Republic is different and the results may vary.

One major limitation of this study is the fact that the prevalence of asthma in one specific geographic region, i.e. Santo Domingo, cannot be interpreted as the prevalence of asthma in the Dominican Republic. Consequently, multiple studies across different rural and urban regions should be performed to improve the accuracy of the prevalence noted.

## Conclusions

The study found that approximately 22.0% of children aged three to 11 years in Santo Domingo suffered from asthma. Age, birth order, family history of asthma and allergy, and exposure to tobacco smoke at home were all associated with the prevalence of asthma. No relationship was found between genders, source of fuel used at home, exposure to a pet and the prevalence of asthma. The higher prevalence of 22.0% corresponded to the same range noted in different studies carried out across different Latin American countries.
